# Rapid diagnosis of membranous nephropathy based on serum and urine Raman spectroscopy combined with deep learning methods

**DOI:** 10.1038/s41598-022-22204-1

**Published:** 2023-02-28

**Authors:** Xueqin Zhang, Xue Song, Wenjing Li, Cheng Chen, Miriban Wusiman, Li Zhang, Jiahui Zhang, Jinyu Lu, Chen Lu, Xiaoyi Lv

**Affiliations:** 1 People’s Hospital of Xinjiang Uygur Autonomous Region, Urumqi, 830001 China; 2 College of Software, Xinjiang University, Urumqi, 830046 China; 3 Xinjiang Medical University, Urumqi, 830054 China; 4 The First Affiliated Hospital of Xinjiang Medical University, Urumqi, 830011 China

**Keywords:** Data processing, Machine learning, Optical spectroscopy, Nephritis

## Abstract

Membranous nephropathy is the main cause of nephrotic syndrome, which has an insidious onset and may progress to end-stage renal disease with a high mortality rate, such as renal failure and uremia. At present, the diagnosis of membranous nephropathy mainly relies on the clinical manifestations of patients and pathological examination of kidney biopsy, which are expensive, time-consuming, and have certain chance and other disadvantages. Therefore, there is an urgent need to find a rapid, accurate and non-invasive diagnostic technique for the diagnosis of membranous nephropathy. In this study, Raman spectra of serum and urine were combined with deep learning methods to diagnose membranous nephropathy. After baseline correction and smoothing of the data, Gaussian white noise of different decibels was added to the training set for data amplification, and the amplified data were imported into ResNet, AlexNet and GoogleNet models to obtain the evaluation results of the models for membranous nephropathy. The experimental results showed that the three deep learning models achieved an accuracy of 1 for the classification of serum data of patients with membranous nephropathy and control group, and the discrimination of urine data was above 0.85, among which AlexNet was the best classification model for both samples. The above experimental results illustrate the great potential of serum- and urine-based Raman spectroscopy combined with deep learning methods for rapid and accurate identification of patients with membranous nephropathy.

## Introduction

Membranous nephropathy, a relatively common glomerular disease, is a major cause of the high prevalence of clinical nephrotic syndrome^1^. Approximately 35–47% of patients with persistent nephrotic syndrome develop renal failure and uremia^2–4^. Xinjiang is a region with a high prevalence of primary glomerular diseases, among which the number of patients with membranous nephropathy (MN) has been increasing in recent years and is gradually becoming younger^5^. Early diagnosis and timely treatment of membranous nephropathy can effectively reduce the deterioration rate of the disease and improve the prognosis^6^. Renal biopsy is the gold standard for the diagnosis of membranous nephropathy at this stage^7^, and this method is invasive and its accuracy depends to a certain extent on the experience of the physician^8^. The remaining common tests such as ultrasonography^9^, light microscopy^10^ and electron microscopy^11^ have disadvantages such as being expensive, time-consuming and susceptible to environmental factors, so there is an urgent need to find a diagnostic method that is simple, inexpensive and accurate and noninvasive^12^.

Raman spectroscopy is an optical detection technique based on inelastic scattering of light, which has the advantages of easy operation, short measurement time, low diagnostic cost and high sensitivity, while the technique provides rich chemical and molecular information for fast, simple and non-invasive analysis of diseases^13,14^. It is widely used in the field of biomedicine and disease diagnosis^15–17^. It has been investigated that body fluids contain biomarkers for a variety of clinical diseases and can be used as a reference for disease diagnosis^18–21^, and Raman spectroscopy based on serum and urine has been used with good results in the diagnosis of a variety of diseases such as breast cancer^22^, cervical cancer^23^, lung cancer^24^ and esophageal cancer ^25^. In recent years, significant progress has been made in the study of Raman spectroscopy applied to the diagnosis of kidney diseases. Jeyse et al. identified potential biomarkers in samples that could cause kidney failure based on Raman spectroscopy of urine and assessed the degree of risk of the samples developing kidney failure^26,27^. Cassiano et al. developed a model for diagnosing kidney-related diseases based on urine samples that can be used to predict future Maurício et al. distinguished between patients with chronic kidney disease and healthy subjects based on serum Raman spectroscopy combined with a principal component analysis (PCA) classification method and obtained a 95% classification accuracy. The aforementioned study showed that the use of Raman spectroscopy for the diagnosis of renal diseases can improve the efficiency and accuracy of diagnosis. It has been shown that as the disease progresses, the levels of certain biomarkers in human serum and urine change, and these changes are potentially associated with the disease and may be useful for its diagnosis and treatment^28^. Sharan et al. uesd dual density dual tree complex wavelet transform to remove Raman spectral noise and spikes^29^. Sanaeifar A et al. used confocal Raman microscopy to spatiotemporal analyze cellular biopolymers on tea plants infected with leaf blight^30^. In this study, we performed classification experiments based on Raman spectroscopy data of serum and urine samples from patients with membranous nephropathy and healthy controls to discover the relationship between changes in substance content between serum and urine while achieving accurate identification of membranous nephropathy.

Machine learning is a subfield of artificial intelligence (AI) and is often applied in fields such as spectral data-assisted medical diagnosis^31^. Traditional machine learning algorithms are highly interpretable and have short training time^32^, but they are not suitable for data with large sample size, high feature dimensionality and strong similarity, and require a lot of pre-processing work on the data to achieve better classification results^33^. The development of neural network models has provided more possibilities for analyzing and processing complex data^34^, and deep neural networks have been designed to improve the shortcomings of traditional machine learning methods^35^, and the network structure can be built according to the characteristics of the data set, making the model more suitable for processing complex and diverse data^36,37^. ResNet introduces a residual module to solve the problem of gradient disappearance with depth deepening^38^. AlexNet uses ReLU as the activation function to avoid overfitting and speed up convergence^39^, and GoogleNet introduces the inception module to improve the training effect by extracting more features with the same amount of computation^40^. All the above three models are optimized and improved to address the shortcomings of traditional machine models to facilitate learning and discrimination of complex data^41^. The above neural networks can approximate the realistic correlations as much as possible, although they cannot completely find the functional relationship between inputs and outputs^42^. In addition, deep learning models have higher fault tolerance and adaptability compared with traditional machine learning techniques, and show higher efficiency and accuracy in processing augmented large sample data, which have greater potential for development and application in future research^43^.

In this study, the diagnosis of membranous nephropathy was performed for the first time based on serum and urine Raman spectral data. The collected serum and urine Raman spectral data were preprocessed and divided into training and test sets. To improve the learning effect of the neural network, the data were expanded by Gaussian white noise data augmentation method and combined with deep learning such as AlexNet, ResNet and GoogleNet frameworks to establish diagnostic models, and to achieve better classification effects, this experiment fine-tuned the above three deep learning algorithms. The classification accuracy of all three classification models reached 1 for serum samples and more than 0.85 for urine samples. The substances corresponding to important features in the samples were analyzed based on the classification results to explore the potential relationship between the changes in substance content in the two body fluid samples. In addition, machine learning algorithms KNN and LDA were selected for comparison experiments, and the accuracy rates were lower than the three deep learning models selected in this paper, which further proved the superiority of the method in this paper for the classification of membranous nephropathy and provided a reference for future research on the diagnosis of nephropathy using deep learning models.

## Materials and methods

### Experimental materials

A total of 73 urine samples were collected in this experiment, including 35 MN patient samples and 38 healthy urine samples; a total of 75 serum samples were collected, including 43 MN patient samples and 32 healthy serum samples. Firstly, the collected serum samples were placed in a refrigerator at 4 °C for 30 min, and the Raman spectral signal of the serum was started to be collected when the serum was thawed. All samples were obtained from the Department of Nephrology, Xinjiang People's Hospital.

### Raman spectral data acquisition

A 15-μL drop of serum was removed onto aluminum foil using a pipette, dried at room temperature and then its Raman signal was measured directly. A high-resolution confocal Raman spectrometer (LabRAM HR Evolution, gora Raman spectroscopy, ideaoptics, China) with a YAG laser at excitation wavelength of 785 nm, an objective lens of 10 × , an integration time of 15 s, and a laser power of 160 mW was used to set the acquisition method to continuous acquisition. The Raman spectra of serum samples in the range of 500–2000 cm^-1^ were measured, and three spectral signals were recorded from different positions of each sample. A total of 35 × 3 urine data were obtained from MN patients and 38 × 3 from healthy controls; 43 × 3 serum data were obtained from MN patients and 32 × 3 from healthy controls. Since the differences between the three data from the same sample were small, the data were averaged for the three data from the same sample and then trained for data amplification and classification.

### Data pre-processing

**Figure 1:**
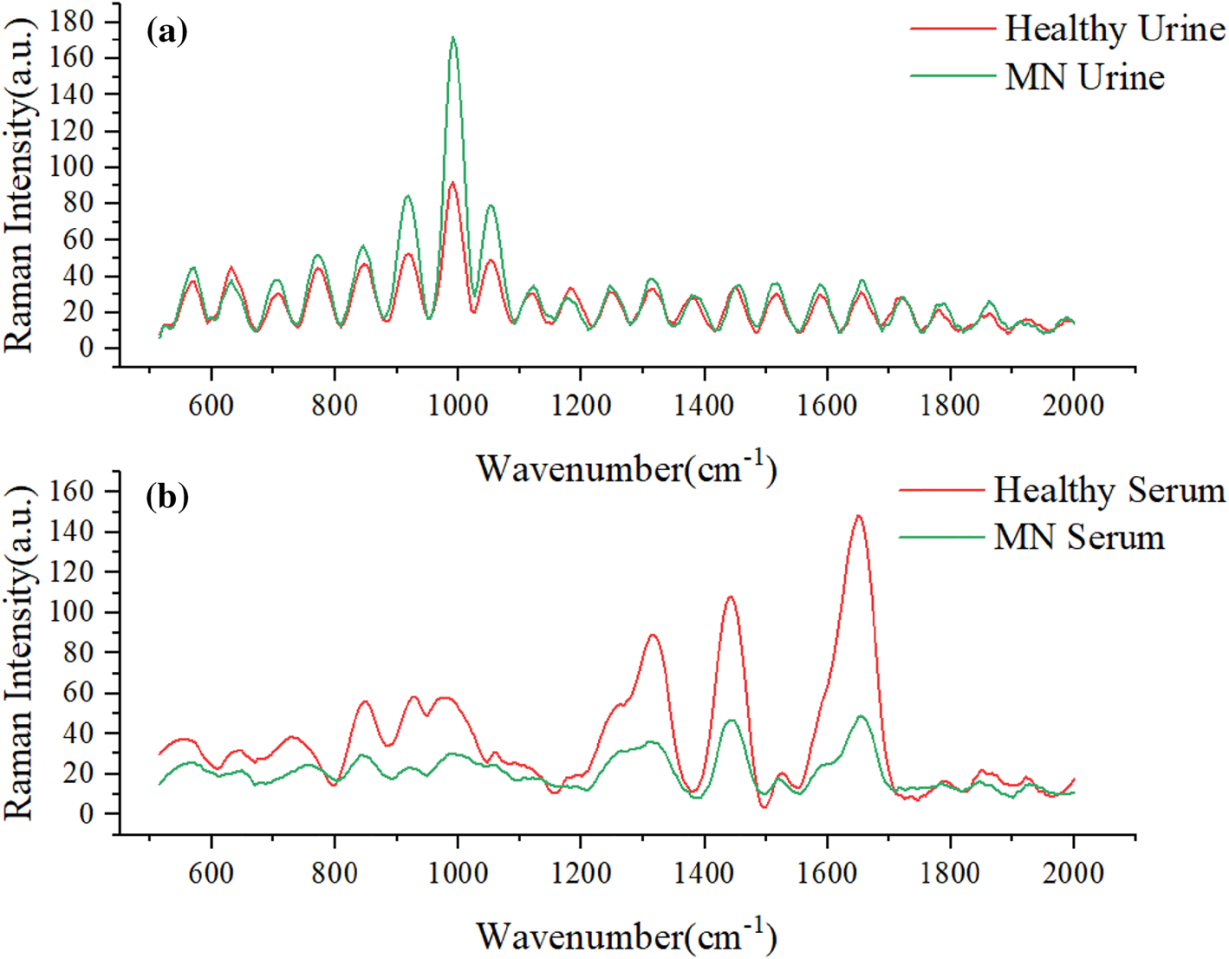
(**a**) Average spectra of urine and healthy samples from MN patients (**b**) Average spectra of serum and healthy samples from MN patients.

As shown in Fig. 1, there is no obvious Raman absorption peak in the range of 2000–4000 cm^−1^, so the serum and urine Raman spectra in the range of 500–2000 cm^−1^ were used in this experiment for biomedical research. Since the raw serum Raman spectra collected by the spectrometer contained noise and fluorescence background, in order to extract the Raman signal accurately and obtain more effective information, the airPLS method was used to perform baseline calibration of the serum and urine Raman spectral data in this paper. After baseline calibration of the raw data, origin 2018(http://www.winwin7.com/soft/51322.html) software was used to smooth the serum sample data using polynomial (Savitzky-Golay) with 20 smoothing points, and MATLAB R2021a(https://ww2.mathworks.cn/products/matlab.html) was used to smoothen the urine sample data with a smoothing window of 9. The average spectra of the two sample data after baseline calibration and smoothing are shown in Fig. 1. The urine and serum samples were divided into training and test sets according to diseased and healthy as 7:3, respectively, and then Gaussian white noise was added to the training set for data augmentation.

### Data enhancement and cross-validation

The training effect of deep learning models improves with the increase of sample size in a certain range, and the large sample size can prevent the occurrence of overfitting and improve the generalization ability of the model to a certain extent. By comparing the existing data control augmentation methods, this study selects the Gaussian white noise data augmentation method to expand the data set. The pre-processed data were divided into training and test sets, and the sample size was expanded to five times the original size by adding five different decibels of Gaussian white noise of 16, 20, 24, 28 and 32 dBW to the training set^44–47^.

In order to evaluate the prediction performance of the model, reduce overfitting and obtain as much valid information as possible from the limited data, the model is validated using the five-fold cross-validation method. This method has the advantage of not requiring additional data splitting, which reduces the computational cost while avoiding data waste.

### Model metrics

In this paper, the performance of the model is evaluated using the true positive rate (TPR), true negative rate (TNR), precision and accuracy, using Eqs. (1)–(4).

1 $$TPR = \frac{TP}{{TP + FN}}$$

2 $$TNR = \frac{TN}{{TN + FP}}$$

3 $$FPR = \frac{FP}{{FP + TN}}$$

4 $$Precision = \frac{TP}{{TP + FP}}$$

5 $$Accuracy = \frac{TP + TN}{{TP + FP + FN + TN}}$$

In addition, ROC curves were plotted with TPR as the vertical coordinate and false positive rate (FPR) as the horizontal coordinate, and AUC values were calculated to comprehensively assess the model performance (Table 1).

**Table 1:** Model evaluation index.

Predict value	Actual value	
	Positive	Negative
Positive	TP	FP
Negative	FN	TN

Note: (True Positive (TP), False Positive (FP), False Negative (FN), True Negative (TN)).

### Ethics approval

This study has been approved from the Cancer Affiliated Hospital of Xinjiang Medical University (in these studies). After obtaining the patient's consent, the patient signs the "Informed Consent Form for Sample Retention at Xinjiang Cancer Hospital of Xinjiang Medical University", which states that "the specimens will be retained only for scientific research in the prevention and treatment of diseases and to reserve important resources for the research and development of medical science and technology. Without prejudice to diagnosis and treatment, tissue specimens will be retained from biopsies or surgical resections, and blood specimens will be retained in 3–10 ml only." The hospital will only retain disease-related specimens after helping the patient understand the consent form and obtaining your consent or that of an authorized person.

### Informed consent

Informed consent was obtained from all participants prior to participating in the interview study. All methods were carried out in accordance with relevant guidelines and regulations (e.g. Helsinki guidelines).

## Results

### Spectral analysis

**Figure 2:**
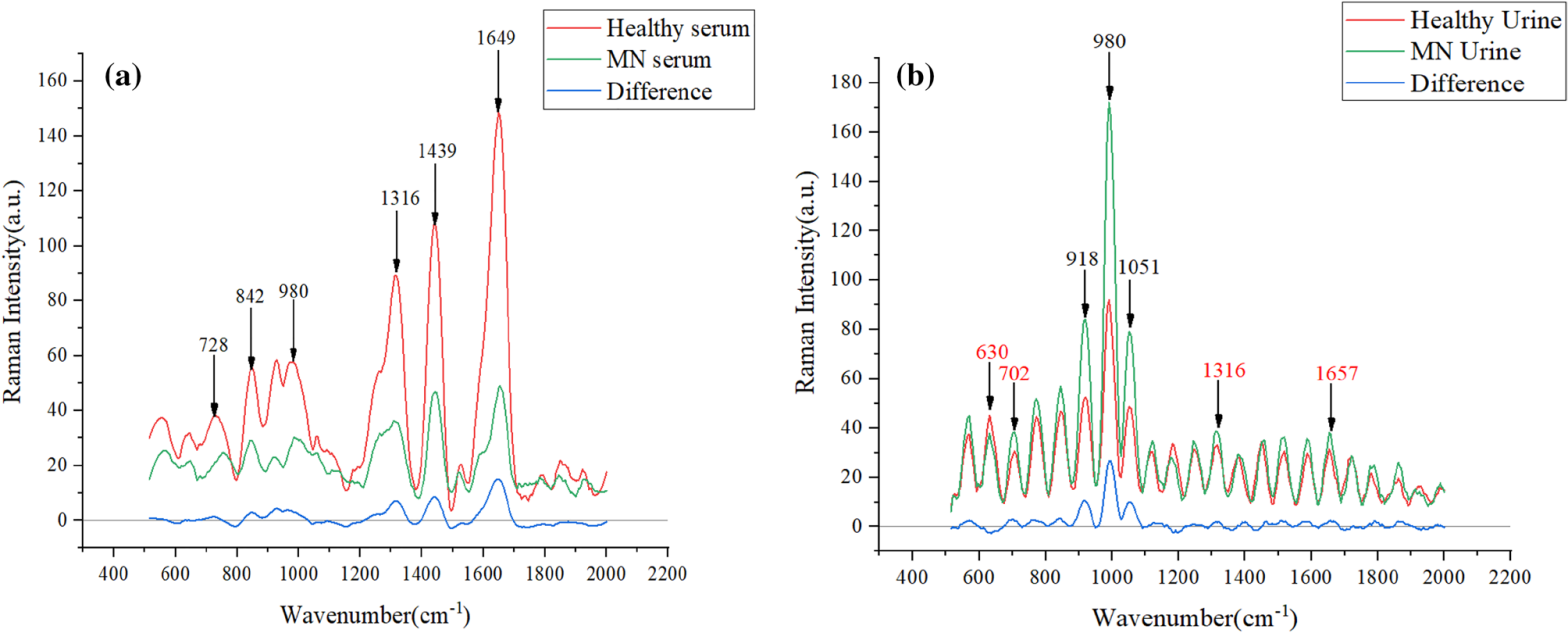
(**a**) Average spectra of serum and control group in membranous nephropathy (**b**) Average spectra of urine and control group in membranous nephropathy.

Figure 2a shows the absorbance of the six peaks with large differences in the serum spectrum, with large peak differences at 728, 842, 980, 1316, 1439 and 1650 cm^−1^; Fig. 2b shows the absorbance of the six peaks with large differences in the urine Raman spectrum, with large peak differences at 630, 918, 980, 1051, 1316 and 1657 cm^−1^. There are large peak differences, especially at 918, 980 and 1051 cm^−1^. These peak differences represent biomolecular differences between patients and control subjects in vivo and can be used as a theoretical basis for disease classification.

In Table 2, the Raman shifts corresponding to the characteristic peaks and their attribution information are listed^48,49^. Combined with Table 2, the glycerol content in the urine of patients with membranous nephropathy is slightly higher. 728 cm^−1^ represents C–C stretch and proline, 842 cm^−1^ represents glucose, 918 cm^−1^ represents proline, strong proline and glycogen, 980 cm^−1^ is protein, 1051 cm^-1^ is lipid, 1316 cm^−1^ is guanine, 1439 cm^−1^ indicates a bent deformation of CH_2_ cm^-1^, three amides at 1650 cm^−1^. The difference in these levels indicates a change in the composition of substances in the serum and urine of patients with membranous nephropathy, resulting in fewer amino acids, guanines, and proteins in patients than in normal subjects^50^.

**Table 2:** Location and substance assignment of characteristic peaks in Raman spectra.

Serum spectral wavenumber	Corresponding substance	Urine spectral wavenumber	Corresponding substance
728	C–C stretching, proline (collagen assignment)	630	Glycerol
842	Glucose	918	Proline, hydroxyproline Glycogen and lactic acid
980	C–C stretching b-sheet (proteins) CH bending (lipids)	980	C–C stretching b-sheet (proteins) CH bending (lipids)
1316	Guanine (B,Z-marker)	1051	Lipids
1439	CH_2_ bending mode in normal tissueCH_3_, CH_2_ deformation CH_2_ scissoring CH_2_ deformation in normal breast tissue	1316	Guanine (B,Z-marker)
1650	(C=C) Amide I Protein amide I absorption Amide I	1657	Fatty acids Amide I (collagen assignment) Triglycerides (fatty acids)

Membranous nephropathy (MN) is a common cause of nephrotic syndrome in adults, and patients usually present with severe hypoproteinemia, which was concluded in the pathogenesis analysis^51^, so that the protein content becomes low in serum samples. Hypoxanthine–guanine phosphoribosyltransferase converts guanine to guanosine 5' monophosphate in order to remedy normal purines when renal function is impaired^52^. Therefore, a decrease in serum guanine levels can occur. In addition, supplementation with amino acids such as proline is effective in patients with kidney disease, which may be related to the reduced amino acid levels in the patient^53^.

In the urine spectrogram, the biomarker corresponding to the position of the largest difference in the Raman peak at 980 cm^−1^ is protein, and the increase in protein in the urine of MN patients correlates with the characteristic pattern of glomerular damage, a phenomenon that also corresponds to changes in the substance content^54^. The clinical manifestations of membranous nephropathy are accompanied by hyperlipidemia and glomerular lipid deposition, so the lipid content is increased^55^. In MN patients with impaired renal function, elevated uric acid occurs, and when guanine content increases, it leads to uric acid deposition in the organism^56^.

### Model design

**Figure 3:**
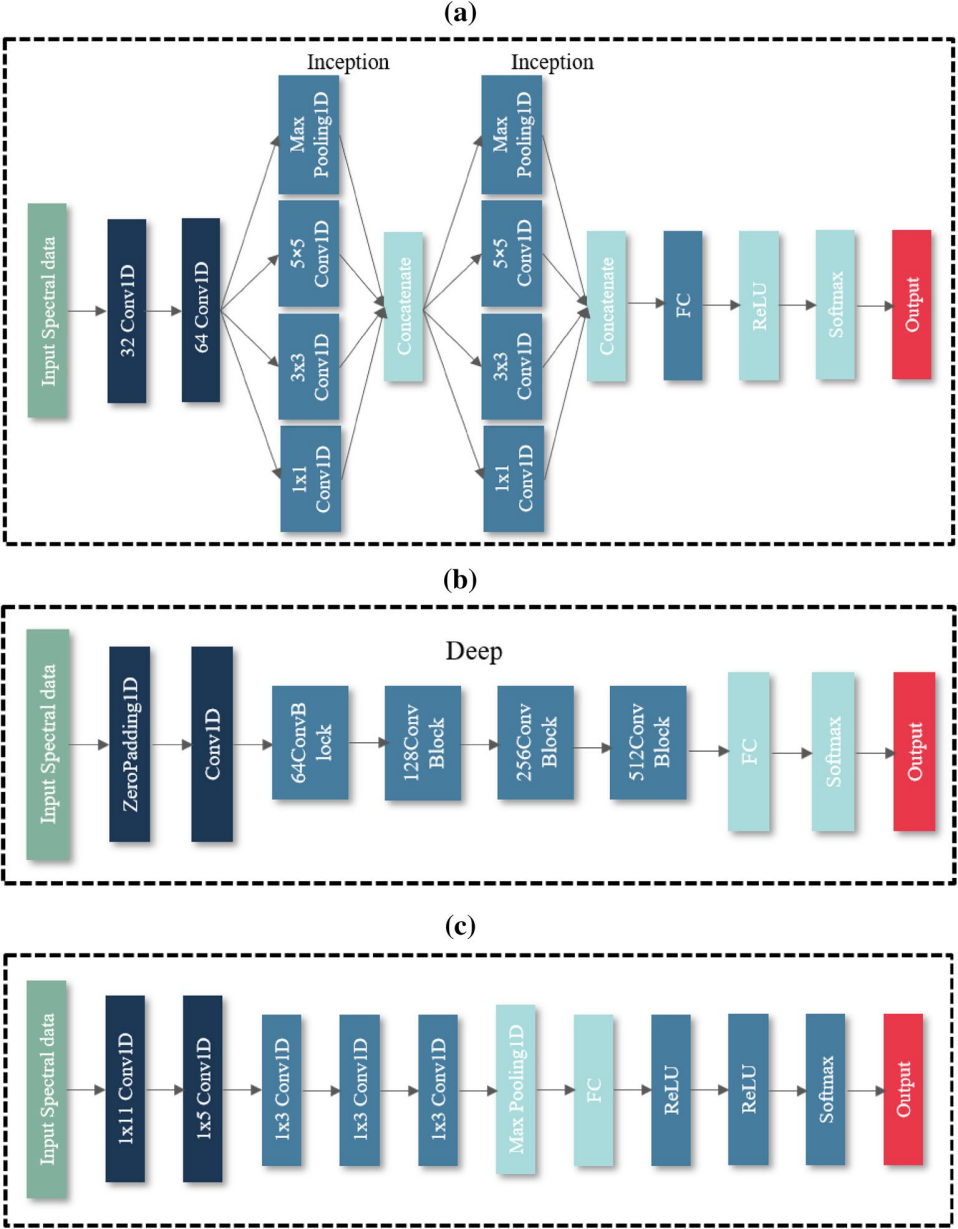
(**a**) GoogleNet network structure (**b**) ResNet network structure (**c**) AlexNet network structure.

In this paper, we choose to use ResNet, AlexNet and GoogleNet deep models and fine-tune the network structure according to the data characteristics, and the structure of each neural network model is shown in Fig. 3.

Figure 3A shows a schematic diagram of the GoogleNet network structure, and by introducing the Inception module, using a 1 × 1 convolution to lift and lower the dimension, and performing simultaneous convolution and reaggregation at multiple dimensions^57^, the model can use resources more efficiently and acquire more features without changing the computational volume. In this study, a GoogleNet network structure containing two initial structural Inception blocks is constructed, and the Inception block is equivalent to a subnetwork containing four channels, which can be controlled by customizing the hyperparameters of each channel to control the model complexity^58^. The filter sizes of the two Inception modules are set to 8 and 16, respectively, and the number of kernels of the two convolutional layers are 32 and 64, respectively, with convolutional kernel sizes of 7 and 3 and step sizes of 2 and 1. The activation functions of the two fully connected layers are chosen as ReLU and Softmax, respectively, with kernel sizes of 256 and 2, respectively.

Figure 3B shows the structure of ResNet network, and ResNet introduces the residual block, which can effectively solve the problem of gradient disappearance and gradient explosion, and solve the degradation problem in the deep network, which allows neurons to be connected in alternate layers and weakens the strong connection between each layer^59^. In this paper, ResNet contains four residual blocks, and the four residual block filter sizes are set to 24, 48, 64 and 128, and the convolution kernel size is all 3 with a step size of 2. Softmax is used as the activation function to output the model processing results.

Figure 3C shows the structure of AlexNet network. Compared with traditional machine learning classification algorithms, AlexNet extends the basic principles of CNN to a deeper and wider network, uses ReLU as the activation function, solves the gradient disappearance problem of Sigmoid, and significantly improves the training speed of the model. In this study, five one-dimensional convolutional layers are constructed with convolutional kernels of 24, 64, 128, 128 and 64, and both fully connected layers have kernels of 128, and Dropout is set to 0.5 to prevent overfitting. All three models use the cross-entropy loss function, and the optimizer is chosen from Adam with 100 iterations and a five-fold cross-validation. This study use Python 3.7(http://www.downza.cn/soft/281667.html) to build the classification model.

### Classification results

**Figure 4:**
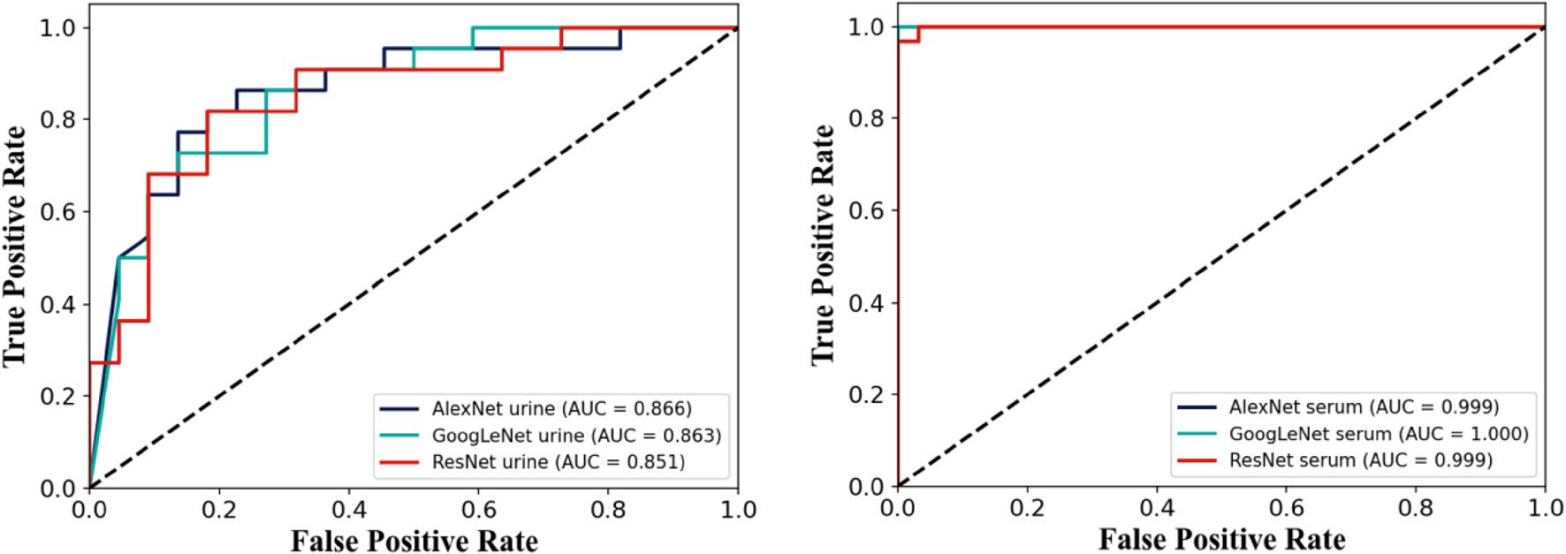
The ROC curves of AlexNet, GoogleNet and ResNet for urine samples on the left, and the ROC curves of AlexNet, GoogleNet and ResNet for serum samples on the right.

Table 3 shows the sensitivity, specificity, AUC values and training time of three different deep models, ResNet, AlexNet and GoogleNet. It can be found that AlexNet based on serum and urine samples has the best training effect and the shortest training time. Figure 4 shows the ROC curves of the models based on urine samples and serum samples, respectively. The classification accuracy of urine samples is lower than that of serum, with ResNet 0.851, AlexNet 0.866, and GoogleNet 0.863, and the classification accuracy of serum samples is higher, close to 1.0. It may be because the substance change of serum samples is larger compared with that of urine samples, and the difference of spectral data is larger thus leading to a good classification effect.

**Table 3:** Neural network model sensitivity, specificity and AUC.

	TPR	TNR	AUC
ResNet(urine)	1.0	0.727	0.87
AlexNet(urine)	0.909	0.818	0.89
GoogleNet(urine)	0.818	0.727	0.86
ResNet(serum)	1.0	1.0	1.0
AlexNet(serum)	1.0	1.0	1.0
GoogleNet(serum)	1.0	1.0	1.0

Supplementary experiments were conducted using traditional machine learning algorithms such as K-neighborhood algorithm (KNN) and linear discriminant analysis (LDA) to classify urine and serum Raman spectral data. As shown in Table 4, the serum Raman spectroscopy dataset showed better classification results, but the classification accuracy of both urine Raman spectroscopy datasets was lower than 85%, so deep neural networks were considered in this study to classify both data to improve the classification accuracy.

**Table 4:** KNN and LDA classification results.

	Accuracy	Recall	F1-score
KNN(urine)	0.81	0.81	0.81
LDA(urine)	0.83	0.84	0.83
KNN(serum)	1.0	1.0	1.0
LDA(serum)	1.0	1.0	1.0

## Discussion

The identification of non-invasive biomarkers of early MN to replace complex and expensive renal biopsy methods is important to prevent the development of nephrotic syndrome and to improve the cure rate of MN patients. The results of spectral analysis showed a correlation between changes in the levels of certain biomarkers in urine and serum samples from MN patients and healthy samples, such as a significant decrease in protein and guanine in serum samples and an increase in urine samples, a change consistent with the clinical presentation of MN patients. The model classified the diseased and healthy controls more accurately according to the significant differences in the levels of these substances, making the model identification results more convincing. The difference in Raman spectral intensity at the peak between patients and normal subjects reflects the difference in the content of biomolecules such as proteins and lipids in the human body, providing a basis for Raman spectroscopy combined with deep learning algorithms to discriminate patients with membranous nephropathy^60^. Although there is variability in the spectral peaks of patients and controls, the small magnitude of this difference makes it difficult to discriminate patients with membranous nephropathy visually from the spectrogram^61^. Therefore, powerful classification models are also needed to achieve rapid and accurate patient identification.

The traditional machine learning model, LDA, maps the data by selecting the projection direction with the best classification performance. Assuming that the classified data conform to Gaussian distribution, LDA follows the principle of minimum intra-class variance and maximum inter-class variance after projection. Because it is a supervised method, LDA may be overfitted by the data itself during the classification process. The prediction results of KNN method are easily affected by noisy data, and when the samples are unbalanced, the classes of new samples are biased toward the classes with the dominant number in the training samples, which may easily lead to prediction errors. In order to make the prediction results more accurate, this study selects deep learning models for further identification of MN patients. All three networks, ResNet, AlexNet and GoogleNet, improved data classification accuracy in different ways while reducing the risk of overfitting. Compared to the three, the ResNet network had the longest training time and was more time-consuming to process large amounts of data, the GoogleNet network was less effective compared to the other two models, and the AlexNet network was optimal with the shortest training time and the highest classification accuracy. The reason for this result may be that the data selected for this experiment are most suitable for the AlexNet network structure and the parameters in the network are set better. The classification results of both samples for membranous nephropathy were better, and this study found the association between the changes in substance content within the two samples while distinguishing more accurately between patients with membranous nephropathy and healthy controls, which provided a basis for the classification of the model and improved the confidence of the classification results.

## Conclusion

In this study, we collected both urine and serum samples based on serum and urine Raman spectra combined with deep learning methods, and were able to distinguish membrane nephropathy samples from healthy controls more accurately, with the accuracy of serum samples close to 100%. In this study, the background noise was firstly removed by airPLS baseline correction of the spectral data, and the important spectral bands were selected, the Gaussian white noise data augmentation improved the robustness of the model, and the five-fold cross-validation increased the reliability of the model classification results. After spectral analysis, it was also found that the same bands existed in the serum and urine spectra of MN patients and controls, with large differences in the peaks at these locations, indicating that the substances corresponding to this band are significant for the classification of membranous nephropathy, and also indicating that analyzing urine and serum simultaneously can enhance the credibility and persuasiveness of the classification results. Among the three deep learning models selected for this study, AlexNet has the best classification effect, with a classification accuracy of 0.89 for urine samples, which is higher than that of traditional machine models, and 1 for serum samples, with the fastest model training speed among the three models. In this study, Raman spectroscopy was used for the first time for the diagnosis of membranous nephropathy, providing a solution for rapid and non-invasive diagnosis of membranous nephropathy, which can effectively improve the diagnostic accuracy and disease cure rate of patients with membranous nephropathy and prevent membranous nephropathy from developing into serious diseases such as nephrotic syndrome or even renal failure.

## Data Availability

The datasets generated and analyzed during the current study are not publicly available due to data privacy laws, but are available from corresponding author on reasonable request.

## References

[1] Tsai S-F, Wu M-J, Chen C-H (2019). Low serum C3 level, high neutrophil-lymphocyte-ratio, and high platelet-lymphocyte-ratio all predicted poor long-term renal survivals in biopsy-confirmed idiopathic membranous nephropathy. Sci. Rep..

[2] van den Brand JAJG, Hofstra JM, Wetzels JFM (2011). Low-molecular-weight proteins as prognostic markers in idiopathic membranous nephropathy. CJASN.

[3] Bose B, Chung EYM, Hong R, Strippoli GFM, Johnson DW, Yang W, Badve SV, Palmer SC (2022). Immunosuppression therapy for idiopathic membranous nephropathy: Systematic review with network meta-analysis. J. Nephrol..

[4] Wang H, Chen C, Tong D, Chen C, Gao R, Han H, Lv X (2021). Serum Raman spectroscopy combined with multiple algorithms for diagnosing thyroid dysfunction and chronic renal failure. Photodiagn. Photodyn. Ther..

[5] Guo, Y., Liu, J.-M., Xu, D., Zhao, Z.-F., Lu, C., Association between PLA2R, HLA-DQA1 gene single nucleotide polymorphism and genetic susceptibility to idiopathic membranous nephropathy in Uygur, (n.d.) 7.

[6] Dabade TS, Grande JP, Norby SM, Fervenza FC, Cosio FG (2008). Recurrent idiopathic membranous nephropathy after kidney transplantation: A surveillance biopsy study. Am. J. Transplant..

[7] Bobart SA, Han H, Tehranian S, De Vriese AS, Roman JCL, Sethi S, Zand L, Andrades Gomez C, Giesen CD, Soler MJ, Bomback AS, Fervenza FC (2021). Noninvasive diagnosis of PLA2R-associated membranous nephropathy: A validation study. CJASN.

[8] Svobodova B, Honsova E, Ronco P, Tesar V, Debiec H (2013). Kidney biopsy is a sensitive tool for retrospective diagnosis of PLA2R-related membranous nephropathy. Nephrol. Dial. Transplant..

[9] Page JE, Morgan SH, Eastwood JB, Smith SA, Webb DJ, Dilly SA, Chow J, Pottier A, Joseph AEA (1994). Ultrasound findings in renal parenchymal disease: Comparison with histological appearances. Clin. Radiol..

[10] Ronco, P., Debiec, H., Gulati, S. Membranous nephropathy, In: D.F. Geary, F. Schaefer (Eds.), Pediatric Kidney Disease, Springer Berlin Heidelberg, Berlin, Heidelberg: pp. 529–546. 10.1007/978-3-662-52972-0_20 (2016).

[11] Yoshimoto K, Yokoyama H, Wada T, Furuichi K, Sakai N, Iwata Y, Goshima S, Kida H (2004). Pathologic findings of initial biopsies reflect the outcomes of membranous nephropathy. Kidney Int..

[12] Lai WL, Yeh TH, Chen PM, Chan CK, Chiang WC, Chen YM, Wu KD, Tsai TJ (2015). Membranous nephropathy: A review on the pathogenesis, diagnosis, and treatment. J. Formos. Med. Assoc..

[13] Krafft C, Popp J (2015). The many facets of Raman spectroscopy for biomedical analysis. Anal. Bioanal. Chem..

[14] Matousek P, Draper ERC, Goodship AE, Clark IP, Ronayne KL, Parker AW (2006). Noninvasive Raman spectroscopy of human tissue in vivo. Appl. Spectrosc..

[15] Motz JT, Gandhi SJ, Scepanovic OR, Haka AS, Kramer JR, Dasari RR, Feld MS (2005). Real-time Raman system for in vivo disease diagnosis. J. Biomed. Opt..

[16] Chen P, Shen A, Zhou X, Hu J (2011). Bio-Raman spectroscopy: A potential clinical analytical method assisting in disease diagnosis. Anal. Methods..

[17] Ellis DI, Goodacre R (2006). Metabolic fingerprinting in disease diagnosis: Biomedical applications of infrared and Raman spectroscopy. Analyst..

[18] Virkler K, Lednev IK (2008). Raman spectroscopy offers great potential for the nondestructive confirmatory identification of body fluids. Forensic Sci. Int..

[19] Bifarin OO, Gaul DA, Sah S, Arnold RS, Ogan K, Master VA, Roberts DL, Bergquist SH, Petros JA, Fernández FM, Edison AS (2021). Machine learning-enabled renal cell carcinoma status prediction using multiplatform urine-based metabolomics. J. Proteome Res..

[20] Tseng Y-J, Huang C-E, Wen C-N, Lai P-Y, Wu M-H, Sun Y-C, Wang H-Y, Lu J-J (2019). Predicting breast cancer metastasis by using serum biomarkers and clinicopathological data with machine learning technologies. Int. J. Med. Inform..

[21] Huang L, Wang L, Hu X, Chen S, Tao Y, Su H, Yang J, Xu W, Vedarethinam V, Wu S, Liu B, Wan X, Lou J, Wang Q, Qian K (2020). Machine learning of serum metabolic patterns encodes early-stage lung adenocarcinoma. Nat. Commun..

[22] Haka AS, Shafer-Peltier KE, Fitzmaurice M, Crowe J, Dasari RR, Feld MS (2005). Diagnosing breast cancer by using Raman spectroscopy. Proc. Natl. Acad. Sci. USA.

[23] Zheng C, Qing S, Wang J, Lü G, Li H, Lü X, Ma C, Tang J, Yue X (2019). Diagnosis of cervical squamous cell carcinoma and cervical adenocarcinoma based on Raman spectroscopy and support vector machine. Photodiagn. Photodyn. Ther..

[24] McGregor HC, Short MA, McWilliams A, Shaipanich T, Ionescu DN, Zhao J, Wang W, Chen G, Lam S, Zeng H (2017). Real-time endoscopic Raman spectroscopy for in vivo early lung cancer detection. J. Biophoton..

[25] Li X, Chen H, Zhang S, Yang H, Gao S, Xu H, Wang L, Xu R, Zhou F, Hu J, Zhao J, Zeng H (2021). Blood plasma resonance Raman spectroscopy combined with multivariate analysis for esophageal cancer detection. J. Biophoton..

[26] Bispo JAM, de Sousa Vieira EE, Silveira L, Fernandes AB (2013). Correlating the amount of urea, creatinine, and glucose in urine from patients with diabetes mellitus and hypertension with the risk of developing renal lesions by means of Raman spectroscopy and principal component analysis. J. Biomed. Opt..

[27] Saatkamp CJ, de Almeida ML, Bispo JAM, Pinheiro ALB, Fernandes AB, Silveira L (2016). Quantifying creatinine and urea in human urine through Raman spectroscopy aiming at diagnosis of kidney disease. J. Biomed. Opt..

[28] Koskimies O, Vilska J, Rapola J, Hallman N (1982). Long-term outcome of primary nephrotic syndrome. Arch. Dis. Child..

[29] Sharan TS, Sharma Sh, Sharma N (2021). Denoising and spike removal from Raman spectra using double density dual-tree complex wavelet transform. J. Appl. Spectrosc..

[30] Li X (2022). A spatial-temporal analysis of cellular biopolymers on leaf blight-infected tea plants using confocal Raman microspectroscopy. Front. Plant Sci..

[31] Sajda P (2006). Machine learning for detection and diagnosis of disease. Annu. Rev. Biomed. Eng..

[32] Kononenko I (2001). Machine learning for medical diagnosis: History, state of the art and perspective. Artif. Intell. Med..

[33] Jordan MI, Mitchell TM (2015). Machine learning: Trends, perspectives, and prospects. Science.

[34] Chattopadhay, A., Sarkar, A., Howlader, P., Balasubramanian, V. N. Grad-CAM++: Generalized gradient-based visual explanations for deep convolutional networks, In: *2018 IEEE Winter Conference on Applications of Computer Vision (WACV)*, IEEE, Lake Tahoe, NV, 2018: pp. 839–847. 10.1109/WACV.2018.00097.

[35] Zhang L, Tan J, Han D, Zhu H (2017). From machine learning to deep learning: Progress in machine intelligence for rational drug discovery. Drug Discov. Today.

[36] Carlini, N., Wagner, D. Towards evaluating the robustness of neural networks, In: 2017 *IEEE Symposium on Security and Privacy (SP)*, IEEE, San Jose, CA, USA, 2017: pp. 39–57. 10.1109/SP.2017.49.

[37] Bishop CM (1994). Neural networks and their applications. Rev. Sci. Instrum..

[38] Szegedy, C., Ioffe, S., Vanhoucke, V., Alemi, A. A. Inception-v4, Inception-ResNet and the Impact of Residual Connections on Learning, (n.d.) 7.

[39] Alom, M. Z., Taha, T. M., Yakopcic, C., Westberg, S., Sidike, P., Nasrin, M. S., Van Esesn, B. C., Awwal, A. A. S., Asari, V. K. The History Began from AlexNet: A Comprehensive Survey on Deep Learning Approaches. 10.48550/ARXIV.1803.01164 (2018).

[40] Fang, T. A Novel computer-aided lung cancer detection method based on transfer learning from GoogLeNet and median intensity projections, In: *2018 IEEE International Conference on Computer and Communication Engineering Technology (CCET)*, IEEE, Beijing, 2018: pp. 286–290. 10.1109/CCET.2018.8542189.

[41] Ertam, F., Ayd, G. Data Classification with Deep Learning using Tensorflow, (n.d.) 4.

[42] Deng L (2014). Deep learning: Methods and applications. FNT Signal Process..

[43] Schmidhuber J (2015). Deep learning in neural networks: An overview. Neural Netw..

[44] Xu Y, Du J, Dai L-R, Lee C-H (2014). An experimental study on speech enhancement based on deep neural networks. IEEE Signal Process. Lett..

[45] Michelsanti, D., Tan, Z.-H. Conditional generative adversarial networks for speech enhancement and noise-robust speaker verification, In: Interspeech, 2017: pp. 2008–2012. 10.21437/Interspeech.2017-1620.

[46] Hao J, Lee T-W, Sejnowski TJ (2010). Speech enhancement using gaussian scale mixture models. IEEE Trans. Audio Speech Lang. Process..

[47] Yue F, Chen C, Yan Z, Chen C, Guo Z, Zhang Z, Chen Z, Zhang F, Lv X (2020). Fourier transform infrared spectroscopy combined with deep learning and data enhancement for quick diagnosis of abnormal thyroid function. Photodiagn. Photodyn. Ther..

[48] Movasaghi Z, Rehman S, Rehman IU (2007). Raman spectroscopy of biological tissues. Appl. Spectrosc. Rev..

[49] Talari ACS, Movasaghi Z, Rehman S, ur Rehman I (2015). Raman spectroscopy of biological tissues. Appl. Spectrosc. Rev..

[50] Waldman M, Austin HA (2012). Treatment of idiopathic membranous nephropathy. JASN..

[51] Glassock R (2003). Diagnosis and natural course of membranous nephropathy. Semin. Nephrol..

[52] Cherian S, Crompton CH (2005). Partial hypoxanthine-guanine phosphoribosyltransferase deficiency presenting as acute renal failure. Pediatr. Nephrol..

[53] Nitta K, Shi S, Nagai T, Kanasaki M, Kitada M, Srivastava SP, Haneda M, Kanasaki K, Koya D (2016). Oral administration of N-Acetyl-seryl-aspartyl-lysyl-proline ameliorates kidney disease in both type 1 and type 2 diabetic mice via a therapeutic Regimen. Biomed. Res. Int..

[54] Bonegio RGB, Fuhro R, Wang Z, Valeri CR, Andry C, Salant DJ, Lieberthal W (2005). Rapamycin ameliorates proteinuria-associated tubulointerstitial inflammation and fibrosis in experimental membranous nephropathy. JASN..

[55] Ohsawa H, Yamabe H, Ozawa K, Fukushi K, Kubota H, Chiba N, Sohma Y, Kanazawa T, Onodera K (1988). Intraglomerular lipid deposition in experimental focal glomerular sclerosis in the Rat. Nephron.

[56] Conger JD (1990). Acute uric acid nephropathy. Med. Clin. North Am..

[57] Szegedy, C., Liu, W., Yangqing Jia, P., Sermanet, S., Reed, D., Anguelov, D., Erhan, V., Vanhoucke, Rabinovich, A. Going deeper with convolutions, In: *2015 IEEE Conference on Computer Vision and Pattern Recognition (CVPR)*, IEEE, Boston, MA, USA, pp. 1–9. 10.1109/CVPR.2015.7298594 (2015).

[58] Tang P, Wang H, Kwong S (2017). G-MS2F: GoogLeNet based multi-stage feature fusion of deep CNN for scene recognition. Neurocomputing.

[59] Wu Z, Shen C, van den Hengel A (2019). Wider or deeper: Revisiting the ResNet model for visual recognition. Pattern Recogn..

[60] Dingari NC, Horowitz GL, Kang JW, Dasari RR, Barman I (2012). Raman spectroscopy provides a powerful diagnostic tool for accurate determination of albumin glycation. PLoS ONE.

[61] Flores-Guerrero, J. L., Muñoz-Morales, A., Narea-Jimenez, F., Perez-Fuentes, R., Torres-Rasgado, E., uiz-Vivanco, G., Gonzalez-Viveros, N., Castro-Ramos, J. Novel assessment of urinary albumin excretion in type 2 diabetes patients by Raman spectroscopy, **11**, (2020). 10.3390/diagnostics10030141 PMC7151048 32138353

